# Association of thyroid function with abnormal lipid metabolism in young patients with first-episode and drug naïve major depressive disorder

**DOI:** 10.3389/fpsyt.2023.1085105

**Published:** 2023-02-14

**Authors:** Jieqiong Hu, Yunxin Ji, Xiaoe Lang, Xiang-Yang Zhang

**Affiliations:** ^1^Department of Psychosomatic Medicine, Ningbo First Hospital, Ningbo, Zhejiang, China; ^2^Department of Psychiatry, First Hospital of Shanxi Medical University, Taiyuan, China; ^3^CAS Key Laboratory of Mental Health, Institute of Psychology, Chinese Academy of Sciences, Beijing, China; ^4^Department of Psychology, University of Chinese Academy of Sciences, Beijing, China

**Keywords:** major depressive disorder, lipid metabolism, thyroid hormones, first-episode, HAMD score

## Abstract

**Introduction:**

Abnormal lipid metabolism in patients with major depressive disorder (MDD) has received increasing attention. The coexistence of MDD and abnormal thyroid function has been intensively studied. Moreover, thyroid function is closely related to lipid metabolism. The aim of this study was to investigate the relationship between thyroid function and abnormal lipid metabolism in young patients with first-episode and drug naïve (FEDN) MDD.

**Methods:**

A total of 1,251 outpatients aged 18–44 years with FEDN MDD were enrolled. Demographic data were collected, and lipid and thyroid function levels were measured, including total cholesterol (TC), triglycerides (TG), low-density lipoprotein cholesterol (LDL-C), high-density lipoprotein cholesterol (HDL-C), thyroid stimulating hormone (TSH), free triiodothyronine (FT3), free tetraiodothyronine (FT4), anti-thyroglobulin antibody (TG-Ab), and anti-thyroid peroxidase antibody (TPO-Ab). The Hamilton Rating Scale for Depression (HAMD), Hamilton Anxiety Rating Scale (HAMA), and Positive and Negative Syndrome Scale (PANSS) positive subscale were also assessed for each patient.

**Results:**

Compared with young MDD patients without comorbid lipid metabolism abnormalities, patients with comorbid lipid metabolism abnormalities had higher body mass index (BMI) values, HAMD score, HAMA score, PANSS positive subscale score, TSH levels, TG-Ab levels, and TPO-Ab levels. Binary logistic regression analysis showed that TSH level, HAMD score and BMI were risk factors for abnormal lipid metabolism. TSH levels were an independent risk factor for abnormal lipid metabolism in young MDD patients. Stepwise multiple linear regression showed that both TC and LDL-C levels were positively correlated with TSH levels, HAMD and PANSS positive subscale scores, respectively. HDL-C levels were negatively correlated with TSH levels. TG levels were positively correlated with TSH and TG-Ab levels and HAMD score.

**Discussion:**

Our results show that thyroid function parameters, especially TSH levels, are implicated in abnormal lipid metabolism in young patients with FEDN MDD.

## 1. Introduction

Mental disorders are increasingly becoming a major cause of the global burden of disease, with depression at the top of the list (1). As a major contributor to this burden, major depressive disorder (MDD) accounts for the largest proportion of disability-adjusted years (DALYs) for mental disorders (2), with relatively higher prevalence estimates and disability weights than many other disorders. In recent years, the prevalence and burden of MDD has increased dramatically due to the COVID-19 pandemic (3).

Epidemiological investigations have found that depression is associated with increased morbidity and mortality from cardiovascular disease (4, 5). It is well established that dyslipidemia increases the risk of cardiovascular disease. In particular, elevated levels of total cholesterol (TC), especially low-density lipoprotein cholesterol (LDL-C) (6) and triglycerides (TG) (7), and decreased levels of high-density lipoprotein cholesterol (HDL-C) (8) are strongly associated with the development of cardiovascular atherosclerosis (9). Abnormalities in lipid levels are also quite common in patients with MDD, but the findings are inconsistent. For example, Wei et al. found that patients with first-episode MDD had higher TG and lower HDL-C levels in the Chinese population, while no significant differences were found in LDL-C and TC levels (10). Bharti et al. found that, compared to controls, MDD patients had higher TG levels and lower TC levels, while HDL-C levels were lower in patients older than 40 years (11). Bot et al. found that depressed patients had higher TG levels and lower HDL-C levels, and this association was consistent across age groups (12). These inconsistent findings may be related to factors such as race, age, and medication use. In addition, previous studies have reported that antidepressant medication can improve lipid profiles and thus reduce the risk of comorbid hyperlipidemia in MDD patients (13). Therefore, the current status of abnormal lipid metabolism in MDD patients and related factors need further study.

Abnormal thyroid function is another common comorbidity in patients with MDD. Either hypothyroidism or hyperthyroidism was positively associated with the risk of clinical depression (14, 15). Subclinical hypothyroidism is associated with suicide risk and psychotic symptoms in patients with MDD (16). Thyroid function indicators are associated with suicidal ideation in patients with MDD (17). Previous studies have found an interaction between thyroid function and lipid metabolism. Thyroid dysfunction, particularly hypothyroidism, can lead to the development of hypercholesterolemia. This is mainly due to reduced activity of the low-density lipoprotein (LDL) receptor. This is accompanied by reduced control of the sterol regulatory element binding protein 2 (SREBP-2) by triiodothyronine (T3), which regulates cholesterol biosynthesis by limiting the activity of the degrading enzyme 3-hydroxy-3-methylglutaryl coenzyme a reductase (HMG-CoA) (18). Meanwhile, hypercholesterolemia may be lipotoxic to the pituitary-thyroid axis. Electron microscopy of mice fed a high-cholesterol diet revealed a significant accumulation of lipid droplets, cytoplasmic loss, and mitochondrial degeneration in thyroid follicular cells (19). It was also found that compared with a mice fed a normal diet, mice fed a high cholesterol diet had elevated serum cholesterol level and TSH levels, significantly increased cholesterol levels in the pituitary gland, and changes in the cytoarchitecture of TSH in the anterior pituitary gland (20). However, the relationship between abnormal lipid metabolism and thyroid function in patients with MDD is unclear.

In this study, we purposefully investigated the incidence of abnormal lipid metabolism and its clinical correlates, especially with thyroid function, in Chinese patients with first-episode drug naïve (FEND) MDD. We hypothesized that the prevalence of abnormal lipid metabolism would be increased in young patients with first-episode and drug naïve (FEDN) MDD and correlate with the clinical presentation of MDD. Moreover, thyroid function plays a role in the development of lipid metabolism abnormalities in young patients with FEDN MDD.

## 2. Materials and methods

### 2.1. Subjects

This was a cross-sectional study, approved by the Ethics Committee of the First Hospital, Shanxi Medical University (No. 2016-Y27). All participants signed an informed consent form prior to enrollment.

Participants were recruited consecutively from September 2015 to December 2017 at the psychiatric outpatient department of the First Affiliated Hospital of Shanxi Medical University. Young adults were defined as 18–44 years old (21). All participants met the following inclusion criteria: (1) age 18–44 years, Han ethnicity; (2) elementary school education or above; (3) meeting the diagnostic criteria for MDD in the Diagnostic and Statistical Manual of Mental Disorders, fourth edition (DSM-IV), which was validated by two experienced psychiatrists according to the Chinese version of the Structured Clinical Interview for DSM-IV (SCID); and (4) first-episode without any prior psychotropic medication such as antidepressants, antipsychotics, or anxiolytics. Exclusion criteria included: (1) other psychiatric disorders diagnosed on Axis I; (2) serious medical abnormalities, such as infection, organ dysfunction, cancer, and trauma; (3) drug/alcohol abuse or dependence, except for nicotine; and (4) pregnant or breastfeeding women.

The sample size was determined by using the following formula: *n* = *Z*^2^*p*(1−*p*)/*d*. n, sample size; *Z*, 95% confidence interval, equal to 1.96; *d*, marginal error, equal to 0.05 (5%); and *p*, expected prevalence, equal to 0.5. The estimated sample size was 384 cases. A total of 1,310 outpatients were screened and 59 patients were excluded for the following reasons: refusal to participate in the study (*n* = 15), severe personality disorder (*n* = 11), pregnant or breastfeeding women (*n* = 10), having a substance use disorder (*n* = 6), severe physical illness (*n* = 7), being unable to be interviewed due to an acute clinical condition (*n* = 4), and having other unspecified reasons (*n* = 6). Finally, a total of 1,251 individuals were included in this study. Thus, the sample size in this study was able to provide sufficient power for statistical analysis.

### 2.2. Clinical measures

A self-designed questionnaire was used, which included age, age of onset, sex, marital status, education, duration of illness (months), and body mass index (BMI). BMI was calculated as weight (kg) divided by the square of height (m) (kg/m^2^).

We used the 17-item Hamilton Rating Scale for Depression (HAMD) (22) to assess depressive symptoms. Eight of its items were scored on a 5-point Likert scale from 0 (none) to 4 (severe), and the other 9 items were scored from 0 (none) to 2 (specific description of symptoms or severity).

The 14-item Hamilton Anxiety Rating Scale (HAMA) was used to assess anxiety symptoms, each scored on a 5-point Likert scale from 0 (none) to 4 (severe) (23). Patients with a HAMA total score of 29 or more were considered to have severe anxiety symptoms (24).

The positive subscale of the Positive and Negative Syndrome Scale (PANSS) was used to assess participants’ psychotic symptoms (25). Each item was scored on a 7-point Likert scale from 1 (none) to 7 (extremely severe). Patients were considered to have psychotic symptoms when they scored 15 or more on the PANSS positive subscale (25, 26).

Prior to the study, two raters were simultaneously trained in the use of the aforementioned scales to ensure consistency and reliability of assessment throughout the study. Their inter-rater correlation coefficient was greater than 0.8. Both raters were blinded to the clinical status of each patient.

### 2.3. Biochemical measurements

Fasting blood was collected from each participant between 7:00 AM and 9:00 AM and immediately sent to the clinical laboratory at the hospital. Thyroid function and blood lipid levels were measured on the same morning. Thyroid function included thyroid stimulating hormone (TSH), free triiodothyronine (FT3), free tetraiodothyronine (FT4), anti-thyroglobulin antibody (TG-Ab), and anti-thyroid peroxidase antibody (TPO-Ab). Lipids included TG, TC, HDL-C, and LDL-C.

Participants in this study with TC ≥5.2 mmol/L or TG ≥1.7 mmol/L or LDL-C ≥3.4 mmol/L or HDL-C <1.0 mmol/L were considered to have abnormal lipid metabolism (27, 28).

### 2.4. Statistical analysis

The Kolmogorov–Smirnov one-sample test was used to determine the normality of all continuous variables. Normally distributed continuous variables were expressed as mean ± standard deviation (*M* ± SD), and non-normally distributed variables were expressed as median (quartiles) [*M*(Q1, Q3)]. Categorical variables were expressed as frequencies and percentages. Demographic and clinical variables were compared between groups of young MDD patients with and without comorbid lipid metabolism abnormalities. Analysis of variance (ANOVA) was used for continuous variables and chi-square test for categorical variables. Bonferroni correction was used to adjust for multiple testing. Subsequently, binary logistic regression (Forward: Wald) was performed to analyze the independent factors affecting the comorbid abnormal lipid metabolism in young MDD patients using the presence or absence of comorbid abnormal lipid metabolism as the dependent variable and the statistically significant variables from the univariate analysis as covariates. Finally, Pearson correlation analysis and further stepwise multiple regression analysis were used to examine the correlation between the levels of each lipid and thyroid function parameter and clinical variables, respectively. Bonferroni correction was used to adjust for multiple testing.

Statistical analyses were performed using SPSS (version 25.0) with a two-tailed *p*-value and significance level set at 0.05.

## 3. Results

### 3.1. Prevalence of abnormal lipid metabolism in young FEDN MDD patients

A total of 1,251 individuals were included in this study. The prevalence of abnormal lipid metabolism in young MDD patients was 74.58% (933/1,251). The rates of high TC, high TG, high LDL-C, and low HDL-C were 48.84% (611/1,251), 61.31% (767/1,251), 27.81% (348/1,251), and 21.98% (275/1,251), respectively.

### 3.2. Comparison of demographic and clinical characteristics between young FEDN MDD patients with and without abnormal lipid metabolism

As shown in Table 1, young MDD patients with lipid metabolism abnormalities had higher BMI, HAMD score, HAMA score, PANSS positive subscale score, TSH levels, TG-Ab levels, and TPO-Ab levels compared with patients without lipid metabolism abnormalities. However, the significance of TG-Ab and TPO-Ab failed to pass the Bonferroni correction (Bonferroni-corrected *p* < 0.05/14 = 0.0036). Further binary logistic regression analysis showed that TSH levels (odd ratio = 1.271, 95% CI = 1.183–1.365, Wald = 43.177, *p* < 0.0001), HAMD score (odd ratio = 1.248, 95% CI = 1.177–1.323, Wald = 55.253, *p* < 0.0001), and BMI (odd ratio = 1.088, 95% CI = 1.008–1.174, Wald = 4.716, *p* < 0.0001) were risk factors for abnormal lipid metabolism.

**TABLE 1:** Comparison of demographic and clinical characteristics between young adult FEDN MDD patients with and without abnormal lipid metabolism.

Variable	MDD without abnormal lipid metabolism	MDD with abnormal lipid metabolism	*F*/*X*^2^	*p*-Value
Sample size	318 (25.4%)	933 (74.6%)		
Sex (male, %)	127 (39.9%)	331 (35.5%)	2.033	0.154
Age (years)	28.63 ± 8.17	28.87 ± 8.43	0.191	0.662
Education (%)			0.169	0.982
Primary school	41 (12.9%)	122 (13.1%)		
High school	153 (48.1%)	446 (47.8%)		
University	101 (31.8%)	303 (32.5%)		
Master’s	23 (7.2%)	62 (6.6%)		
Marital status (single, %)	128 (40.3%)	359 (38.5%)	0.314	0.575
Age of onset (years)	28.47 ± 8.09	28.72 ± 8.33	0.220	0.639
Duration of illness (months)	5.58 ± 4.31	5.65 ± 4.0	0.057	0.812
BMI (kg/m^2^)	23.95 ± 1.87	24.48 ± 1.96	17.289	0.000
HAMD	28.50 ± 2.63	30.77 ± 2.79	161.406	0.000
HAMA	19.58 ± 3.23	21.01 ± 3.36	43.595	0.000
PANSS (P sub-scale)	7.70 ± 3.07	8.93 ± 4.26	22.655	0.000
TSH (mIU/L)	3.51 ± 1.92	5.42 ± 2.53	150.239	0.000
A-TG (IU/ml)	59.48 ± 120.68	93.77 ± 239.61	5.995	0.014
A-TPO (IU/ml)	46.57 ± 92.00	75.79 ± 173.37	8.238	0.004
FT3 (pmol/L)	4.86 ± 0.72	4.95 ± 0.737	3.587	0.058
FT4 (pmol/L)	16.64 ± 3.04	16.77 ± 3.10	0.472	0.492

BMI, body mass index; HAMD, Hamilton Rating Scale for Depression; HAMA, Hamilton Anxiety Rating Scale; PANSS (P sub-scale), positive sub-scale of the Positive and Negative Syndrome Scale; TSH, thyroid stimulating hormone; A-TG, anti-thyroglobulin antibody; A-TPO, anti-thyroid peroxidase antibody; FT3, free triiodothyronine; FT4, free tetraiodothyronine.

### 3.3. Correlation of TC, LDL-C, HDL-C, and TG levels and clinical variables

The results of Pearson correlation analysis between the levels of each lipid component and clinical variables are shown in Table 2. TC levels were significantly correlated with age (*r* = 0.070, *p* = 0.013), age at onset (*r* = 0.070, *p* = 0.013), duration of disease (*r* = 0.064, *p* = 0.024), BMI (*r* = 0.060, *p* = 0.033), HAMD score (*r* = 0.569, *p* < 0.001), HAMA score (*r* = 0.291, *p* < 0.001), PANSS positive subscale score (*r* = 0.211, *p* < 0.001), TSH level (*r* = 0.559, *p* < 0.001), TG-Ab level (*r* = 0.098, *p* < 0.001), and TPO-Ab level (*r* = 0.135, *p* < 0.001). However, the significance of age, age at onset, duration of disease, and BMI failed to pass the Bonferroni correction (Bonferroni-corrected *p* < 0.05/15 = 0.0033). Further stepwise multiple linear regression showed that HAMD scores (β = 0.472, *t* = 17.693, *p* < 0.001), TSH levels (β = 0.392, *t* = 16.284, *p* < 0.001), and PANSS positive subscale scores (β = −0.172, *t* = −6.92, *p* < 0.001) were independently associated with TC levels.

**TABLE 2:** Inter-relationships between demographic and clinical variables in young adult patients with FEDN MDD.

Variables	Age	Age of onset	Duration of illness	HAMD	HAMA	P-PANSS	TSH	A-TG	A-TPO	FT3	FT4	BMI	TC	LDL-C	HDL-C	TG
Age	1	0.999**	0.313**	0.034	0.009	-0.042	0.034	0.008	0.063	-0.034	-0.015	0.048	0.070*	0.055	0.008	0.032
Age of onset	0.000	1	0.278**	0.035	0.010	-0.040	0.031	0.009	0.034	-0.033	-0.013	0.045	0.070*	0.053	0.008	0.034
Duration of illness	0.000	0.000	1	0.054	0.020	-0.018	0.141**	-0.018	0.031	-0.069*	-0.009	0.074**	0.064*	0.059*	-0.008	-0.006
HAMD	0.227	0.222	0.056	1	0.597**	0.528**	0.479**	0.092**	0.140**	0.034	0.008	0.061*	0.569**	0.395**	-0.144**	0.151**
HAMA	0.738	0.717	0.490	0.000	1	0.582**	0.321**	0.121**	0.131**	-0.001	0.029	0.025	0.291**	0.205**	-0.089**	0.091**
P-PANSS	0.141	0.156	0.528	0.000	0.000	1	0.341**	0.092**	0.123**	-0.010	0.024	0.027	0.211**	0.134**	-0.104**	0.118**
TSH	0.225	0.267	0.000	0.000	0.000	0.000	1	0.213**	0.274**	0.033	0.033	0.156**	0.559**	0.362**	-0.343**	0.169**
A-TG	0.777	0.746	0.527	0.001	0.000	0.001	0.000	1	0.433**	0.026	-0.022	-0.037	0.098**	0.027	-0.097**	0.095**
A-TPO	0.217	0.233	0.278	0.000	0.000	0.000	0.000	0.000	1	0.000	0.021	-0.012	0.135**	0.069*	-0.101**	0.059*
FT3	0.223	0.246	0.014	0.236	0.981	0.723	0.243	0.357	0.997	1	0.234**	0.019	0.007	-0.024	-0.065*	0.025
FT4	0.599	0.651	0.743	0.790	0.309	0.403	0.241	0.444	0.462	0.000	1	0.037	-0.016	-0.007	-0.032	-0.022
BMI	0.091	0.009	0.091	0.032	0.385	0.338	0.000	0.190	0.679	0.508	0.192	1	0.060*	0.061*	-0.038	0.048
TC	0.013	0.013	0.024	0.000	0.000	0.000	0.000	0.001	0.000	0.818	0.577	0.033	1	0.589**	-0.234**	0.224**
LDL-C	0.053	0.059	0.038	0.000	0.000	0.000	0.000	0.348	0.015	0.387	0.792	0.030	0.000	1	-0.180**	0.000
HDL-C	0.791	0.780	0.780	0.000	0.002	0.000	0.000	0.001	0.000	0.022	0.251	0.182	0.000	0.000	1	-0.137**
TG	0.252	0.232	0.844	0.000	0.001	0.000	0.000	0.001	0.036	0.380	0.433	0.087	0.000	0.986	0.000	1

**p < 0.01; *p < 0.05.

Low-density lipoprotein cholesterol levels were significantly associated with duration of disease (*r* = 0.059, *p* = 0.038), BMI (*r* = 0.061, *p* = 0.030), HAMD score (*r* = 0.395, *p* < 0.001), HAMA score (*r* = 0.205, *p* < 0.001), PANSS positive subscale score (*r* = 0.134, *p* < 0.001), TSH level (*r* = 0.362, *p* < 0.001), and TPO-Ab level (*r* = 0.069, *p* = 0.015). However, the significance of duration of disease, BMI and TPO-Ab level failed to pass the Bonferroni correction (Bonferroni-corrected *p* < 0.05/15 = 0.0033). Further stepwise multiple linear regression showed that HAMD score (β = 0.351, *t* = 10.971, *p* < 0.001), TSH levels (β = 0.239, *t* = 8.257, *p* < 0.001), and PANSS positive subscale score (β = −0.134, *t* = −4.466, *p* < 0.001) were independently associated with LDL-C levels.

High-density lipoprotein cholesterol levels were significantly associated with HAMD score (*r* = −0.144, *p* < 0.001), HAMA score (*r* = −0.089, *p* = 0.002), PANSS positive subscale score (*r* = −0.104, *p* < 0.001), TSH levels (*r* = −0.343, *p* < 0.001), TG-Ab levels (*r* = −0.097, *p* = 0.001), TPO-Ab level (*r* = −0.101, *p* < 0.001), and FT3 level (*r* = −0.065, *p* = 0.022). However, the significance of FT3 level failed to pass the Bonferroni correction (Bonferroni-corrected *p* < 0.05/15 = 0.0033). Stepwise multiple linear regression showed that only TSH levels (β = −0.343, *t* = −12.891, *p* < 0.001) were independently associated with HDL-C levels.

Triglycerides levels were correlated with HAMD score (*r* = 0.151, *p* < 0.001), HAMA score (*r* = 0.091, *p* = 0.001), PANSS positive subscale score (*r* = 0.118, *p* < 0.001), TSH level (*r* = 0.169, *p* < 0.001), TG-Ab level (*r* = 0.095, *p* = 0.001), and TPO-Ab levels (*r* = 0.059, *p* = 0.036). However, the significance of TPO-Ab level failed to pass the Bonferroni correction (Bonferroni-corrected *p* < 0.05/15 = 0.0033). Stepwise multiple linear regression showed that TSH levels (β = 0.112, *t* = 3.478, *p* = 0.001), HAMD scores (β = 0.091, *t* = 2.881, *p* = 0.004), and TG-Ab levels (β = 0.063, *t* = 2.207, *p* = 0.028) were independently associated with TG levels.

## 4. Discussion

To our knowledge, this is the first study in China on the correlation between abnormal lipid metabolism and thyroid function in young patients with FEND MDD. The main findings of this study were that patients with abnormal lipid metabolism had higher HAMD, HAMA, and PANSS positive subscale scores, higher TSH, TG-Ab, TPO-Ab, and BMI levels. In addition, TSH levels were an independent risk factor for comorbid lipid metabolism abnormalities in young patients with FEND MDD.

Our study found that depressive symptoms were more severe in young FEND MDD patients with than without lipid metabolism abnormalities. In addition, HAMD score was significantly correlated with all four lipid components, specifically, positively with TC, LDL-C, and TG levels and negatively with HDL-C levels. Previous studies have shown that the severity of depressive symptoms is associated with lipid metabolism, but with inconsistent results. For example, some studies have found that the severity of depressive symptoms is positively correlated with TG, TC, and LDL-C levels and negatively correlated with HDL-C levels, consistent with the results of our present study (29, 30). Several other studies have found low HDL-C levels to be a predictor of the severity of depressive symptoms in patients with FEDN MDD (31). A positive correlation between TC and LDL-C levels and the severity of depressive symptoms has also been reported (32). In the general population, low HDL-C levels are found to be associated with the development of depressive symptoms (33), while in the general middle-aged population, high HDL-C levels or high TC levels are found to be risk factors for the development of depressive symptoms (34–36). The results of studies on the causal relationship between depression and lipid composition have also been inconsistent. In a Mendelian randomization study of a European population, one result showed a possible causal relationship between TC and depression (37), while another did not find a causal relationship between the two (38). We also found that patients with abnormal lipid metabolism also had higher HAMA score compared to patients without abnormal lipid metabolism. Moreover, HAMA score was positively correlated with TC, LDL-C, and TG levels and negatively correlated with HDL-C levels, which is consistent with the findings of Yan et al. (39), who observed a negative correlation between HAMA score and HDL levels in depressed patients (40). In addition to depressive symptoms and anxiety symptoms, we also found higher PANSS positive subscale score in MDD patients with comorbid lipid metabolism abnormalities. Similarly, psychotic symptoms were positively correlated with TC, LDL-C, and TG levels and negatively correlated with HDL-C, which is consistent with the findings of Wang et al. (41). However, another cross-sectional study showed that MDD patients with comorbid severe anxiety or psychotic symptoms had higher TC, TG, and LDL-C levels and lower HDL-C levels compared to those without comorbidities (42). Taken together, despite the differences in the results of these aforementioned studies, they largely support our findings that clinical features including depressive symptoms, anxiety symptoms, and psychotic symptoms are more pronounced in MDD patients with abnormal lipid metabolism and are significantly associated with lipid levels.

This study also found that young MDD patients with lipid metabolism abnormalities had higher levels of TSH, TPO-Ab, and TG-Ab than patients without lipid metabolism abnormalities. Furthermore, TSH levels were an independent risk factor for abnormal lipid metabolism in young MDD patients. Previous studies have shown that thyroid hormone (TH) and TSH play an important role in dyslipidemia (43–45). TH has contradictory effects on cholesterol absorption and production. TH increases cholesterol synthesis by directly inducing hepatic HMG-COA reductase (HMGCR) expression (46), but TH decreases cholesterol absorption by affecting Niemann-Pick C1-like protein (NPC1L1) in the intestine (47) and increases catabolism by enhancing free fatty acid β-oxidation to increase catabolism (48). TSH can directly affect cholesterol synthesis by upregulating HMGCR expression and activity through the cAMP/PKA/CREB signaling pathway (49), increasing HMGCR mRNA levels (50), and increasing phosphorylated hormone-sensitive lipase (HSL) to increase lipolysis (51). On the other hand, TSH plays an important role in the clearance of LDL and induces PI3K/AKT/SREBP2 and SREBP2/HNF4/cholesterol 7α-hydroxylase (CYP7A1) signaling pathways to inhibit hepatic bile acid synthesis (52). Higher TSH levels are associated with a greater risk of dyslipidemia (53). Furthermore, TSH levels are positively correlated with TC, LDL-C, and TG levels, while the correlation between TSH levels and HDL-C levels is uncertain (54–56). Recent studies have shown that lipids can also adversely affect thyroid function. The thyroid may be one of the target organs for lipotoxicity, and high circulating TG are a potential risk factor for subclinical hypothyroidism (57). Moreover, excessive accumulation of cholesterol may induce thyroid dysfunction (58, 59). Previous studies in depressed patients have found a positive correlation between TSH levels and HDL-C levels (60). In depressed patients with long duration of symptoms, TSH levels were positively correlated with TC and LDL-C levels (61). In this study, TSH levels were positively correlated with TC, TG, and LDL-C levels and negatively correlated with HDL-C levels in FEDN patients with MDD.

In addition, previous studies have reported an association between thyroid autoimmune function and depression, but the results have been inconsistent. Siegmann et al. (62) has predicted that more than 20% of patients with autoimmune hypothyroidism will develop depression annually, suggesting a strong association between thyroid autoimmunity and depression. In contrast, a study by Bode et al. (63) revealed no statistically significant association between autoimmunity (mainly TPO antibody status) and depression. In this study, we found that both TPO-Ab and TG-Ab levels were positively correlated with HAMD score in young FEDN MDD patients. Previous studies have shown an association between thyroid autoimmune antibody status and lipid metabolism, with varying results. Li et al. (64) found that TG-Ab was positively correlated with LDL-C and HDL-C, but not with TC and TG, while TPO-Ab was positively correlated with LDL-C only. Cengiz et al. (65) found that TG-Ab and TPO-Ab were positively correlated with TC, TG, and LDL-C, respectively, and not with HDL-C. Studies also found that TG-Ab was negatively correlated with TG, whereas TPO-Ab was negatively correlated with HDL-C (66, 67). In this study, TG-Ab was found to be positively correlated with TC and TG and negatively correlated with HDL-C in young FEDN MDD patients. TPO-Ab was positively correlated with TC, TG, and LDL-C and negatively correlated with HDL-C. Taken together, these findings suggested a correlation between thyroid autoimmune antibodies and lipid components in MDD patients, while only TG-Ab was independently associated with TG.

This study had several limitations. First, this is a cross-sectional study and cannot demonstrate a causal relationship between abnormal lipid metabolism and thyroid function in young patients with FEDN MDD. Second, although the potential confounders had been adjusted for, we did not exclude factors influencing lipid metabolism, such as dietary structure, exercise status, smoking, and alcohol consumption, which may had partially biased the results. Third, a comprehensive assessment of thyroid status may not be possible due to the lack of imaging data of the thyroid gland. Fourth, we excluded pregnant and lactating women, taking into account different endocrine levels. However, abnormalities of thyroid function and lipid metabolism in MDD patients during pregnancy and postpartum need to be investigated in future studies. Fifth, as this study was conducted in only one general hospital in China, the results of this study may not be generalizable to other settings. Finally, there was no healthy control group in this study. Therefore, this is only a preliminary result.

## 5. Conclusion

In summary, in this study, we found that the comorbid lipid metabolism abnormalities in young patients with FEDN MDD were associated with thyroid function parameters, especially TSH levels, which was an independent risk factor. Moreover, TSH levels were associated with TC, LDL-C, HDL-C, and TG levels in young patients with FEDN MDD. Simultaneously, TG-Ab levels were independently associated with TG levels. In the future, a longitudinal study needs to be designed that includes healthy controls and excludes confounding factors that may affect abnormal lipid metabolism, such as the presence of a high-fat diet, alcohol consumption, and sedentary lifestyle. We also need to refine thyroid imaging for a more comprehensive assessment in order to perform an exhaustive analysis according to different thyroid states. This allows us to explore in greater depth the relationship between thyroid function and abnormal lipid metabolism in MDD patients to further validate the results of this study. Abnormal lipid metabolism in young MDD patients is significantly associated with TSH levels and there is potential for future interventions in TSH to prevent dyslipidemia and thereby reduce the incidence of cardiovascular events.

## Data availability statement

The original contributions presented in this study are included in this article/supplementary material, further inquiries can be directed to the corresponding author.

## Ethics statement

This study was reviewed and approved by the Ethics Committee of the First Hospital, Shanxi Medical University (No. 2016-Y27). All participants signed an informed consent form prior to enrollment.

## Author contributions

JH: conceptualization, methodology, analysis and interpretation of data for the work, and drafting the work. YJ: funding acquisition, resources, and supervision. XL: investigation and data curation. X-YZ: substantial contributions to the conception or design of the work and revising the work critically for important intellectual content. All authors contributed to the article and approved the submitted version.
